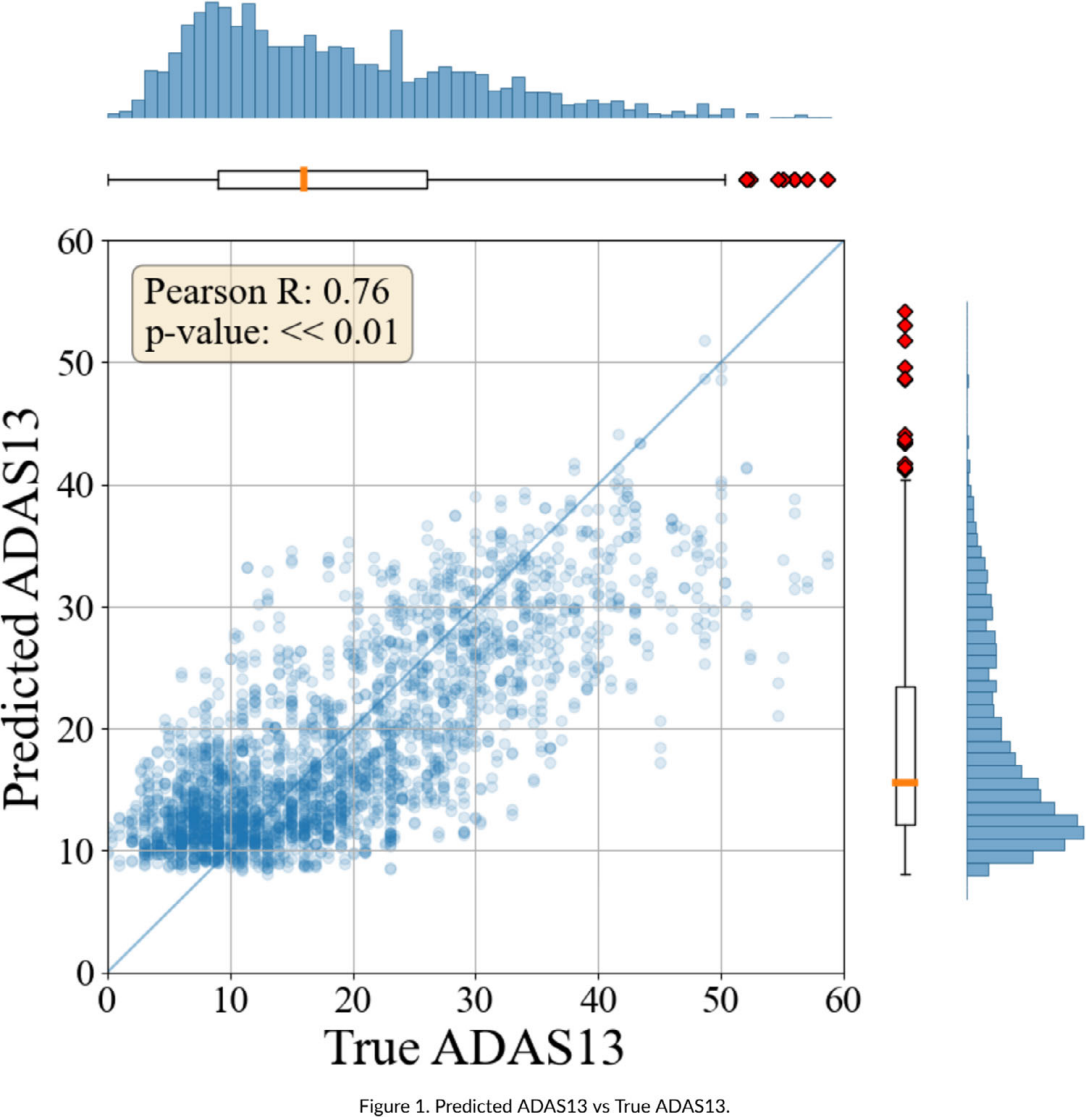# Predicting Alzheimer’s Disease Assessment Scale from T1‐weighted MRIs by Fine‐tuning a Pretrained Deep Learning Model

**DOI:** 10.1002/alz70862_110184

**Published:** 2025-12-23

**Authors:** Reza Rajabli, Mahdie Soltaninejad, D Louis Collins

**Affiliations:** ^1^ McConnell Brain Imaging Centre, Montreal Neurological Institute, McGill University, Montreal, QC Canada

## Abstract

**Background:**

Developing diagnostic and prognostic tools for Alzheimer’s disease is challenging due to clinical variability across stages. While many studies have focused on case–control classification and conversion prediction, fewer have explored MRI‐based prediction of clinical assessment scores, such as the Alzheimer’s Disease Assessment Scale (ADAS), despite its potential for measuring disease severity and aiding prognosis. Deep learning could enhance these predictions, but limited labeled data in Alzheimer’s disease research constrains model training. To address this, we investigated whether a pretrained, robust brain age prediction model could be fine‐tuned to predict clinical scores more effectively.

**Method:**

We built an ensemble (*n* = 5) model to predict brain age from 3D brain MRI. To ensure generalizability, we applied robust preprocessing methods, extensive data augmentation, and regularization techniques, achieving a Mean Absolute Error (MAE) of 3.17 years on average on multiple unseen external test datasets (Rajabli, 2024).

We split 11,041 MRIs from the Alzheimer’s Disease Neuroimaging Initiative (ADNI1, ADNI2, and ADNI‐Go) dataset into a training set (*n* = 5,536), validation set (*n* = 2,815), and test set (*n* = 2,690), ensuring that no subject appeared in more than one set (ADNI is a longitudinal cohort). We then fine‐tuned our model to predict ADAS13 on the training set and evaluated it on the validation and test sets.

**Result:**

In ADNI, the mean ADAS13 score is 17.92 with a standard deviation of 11.42. We achieved a Mean Absolute Error (MAE) of 5.66, 6.46 and 5.90 on the training, validation and test sets, respectively, for predicting ADAS13. The R² score on the test set is 0.58 (*r* = 0.76, *p* << 0.01). Figure 1 displays the scatter plot of predicted ADAS13 versus true ADAS13 values for the test set.

**Conclusion:**

Using only 50% of the available data for training, we introduced a prediction model which generalized well to the test set, demonstrating the robustness of our model. Our approach required less data while achieving superior results compared to previous methods (such as Bhagwat 2019), paving the way for training more generalizable networks with limited data—a crucial factor for medical imaging datasets.